# Structure and Dynamics of Single-isoform Recombinant Neuronal Human Tubulin*[Fn FN3]^♦^

**DOI:** 10.1074/jbc.C116.731133

**Published:** 2016-04-25

**Authors:** Annapurna Vemu, Joseph Atherton, Jeffrey O. Spector, Agnieszka Szyk, Carolyn A. Moores, Antonina Roll-Mecak

**Affiliations:** From the ‡ Cell Biology and Biophysics Unit, NINDS, and; ¶ Biophysics Center, NHLBI, National Institutes of Health, Bethesda, Maryland 20892 and; the § Institute of Structural and Molecular Biology, Department of Biological Sciences, Birkbeck College, University of London, London WC1E, United Kingdom

**Keywords:** cryo-electron microscopy, cytoskeleton, microscopy, microtubule, tubulin, dynamic instability, engineered tubulin, microtubule dynamics, recombinant tubulin, tubulin isoform, microtubule structure, cryo-EM, post-translational modification

## Abstract

Microtubules are polymers that cycle stochastically between polymerization and depolymerization, *i.e.* they exhibit “dynamic instability.” This behavior is crucial for cell division, motility, and differentiation. Although studies in the last decade have made fundamental breakthroughs in our understanding of how cellular effectors modulate microtubule dynamics, analysis of the relationship between tubulin sequence, structure, and dynamics has been held back by a lack of dynamics measurements with and structural characterization of homogeneous isotypically pure engineered tubulin. Here, we report for the first time the cryo-EM structure and *in vitro* dynamics parameters of recombinant isotypically pure human tubulin. α1A/βIII is a purely neuronal tubulin isoform. The 4.2-Å structure of post-translationally unmodified human α1A/βIII microtubules shows overall similarity to that of heterogeneous brain microtubules, but it is distinguished by subtle differences at polymerization interfaces, which are hot spots for sequence divergence between tubulin isoforms. *In vitro* dynamics assays show that, like mosaic brain microtubules, recombinant homogeneous microtubules undergo dynamic instability, but they polymerize slower and have fewer catastrophes. Interestingly, we find that epitaxial growth of α1A/βIII microtubules from heterogeneous brain seeds is inefficient but can be fully rescued by incorporating as little as 5% of brain tubulin into the homogeneous α1A/βIII lattice. Our study establishes a system to examine the structure and dynamics of mammalian microtubules with well defined tubulin species and is a first and necessary step toward uncovering how tubulin genetic and chemical diversity is exploited to modulate intrinsic microtubule dynamics.

## Introduction

Microtubules cycle stochastically between periods of polymerization and depolymerization, *i.e.* they exhibit “dynamic instability” (1). This behavior is crucial in cell division, motility, and differentiation. Despite the discovery of dynamic instability more than 30 years ago (1) and fundamental breakthroughs in our understanding of microtubule dynamics modulation by cellular effectors (2, 3), analysis of the relationship between tubulin sequence, structure, and dynamics has been held back by a lack of structural and *in vitro* dynamics data with homogeneous isotypically pure engineered tubulin. Eukaryotes have multiple tubulin genes (humans have eight α- and eight β-tubulin isotypes) with tissue-specific distributions (4). Some microtubules are isotype mixtures, and others are formed from a predominant single isotype (5). Moreover, tubulin is subject to abundant and chemically diverse post-translational modifications that include acetylation, detyrosination, phosphorylation, glutamylation, glycylation, and amination (6, 7). Virtually all biochemical studies have used tubulin purified from mammalian brain tissue through multiple cycles of *in vitro* depolymerization and polymerization (8). Although tubulin is abundant in this source, the resulting material is highly heterogeneous, being composed of multiple tubulin isotypes bearing chemically diverse and abundant post-translational modifications (9–11). More than 22 different charge variants are repolymerized in random fashion for *in vitro* polymerization assays (12). Thus, microtubules used for *in vitro* dynamics assays have been mosaic, with random distributions of isoforms and post-translational modifications. Moreover, this purification procedure selects tubulin subpopulations that polymerize robustly while discarding those that do not. Efforts to reduce metazoan tubulin heterogeneity exploited differences in isoform compositions between various tissues or cell lines (*e.g.* avian erythrocytes (13) and HeLa cells (14)) or the use of isoform-specific antibodies for immunopurification (15). However, neither of these approaches yielded homogeneous single-isoform tubulin. Here, we report for the first time the expression and purification of recombinant isotypically pure unmodified human tubulin competent for *in vitro* dynamics assays and report its dynamic parameters as well as cryo-EM structure at 4.2 Å resolution. We find that isotypically pure unmodified α1A/βIII-tubulin exhibits subtle differences in dynamics when compared with heterogeneous brain tubulin, consistent with the small conformational rearrangements at tubulin polymerization interfaces revealed by our near-atomic resolution structure of α1A/βIII microtubules. Our study establishes a system to examine the structure and dynamics of mammalian microtubules with well defined α and β-tubulin species and is a first and necessary step toward exploring the biophysical correlates between sequence, structure, and dynamics for mammalian microtubules.

## Experimental Procedures

### 

#### 

##### Expression and Purification of Human Recombinant Tubulin Constructs

**FIGURE 1.**
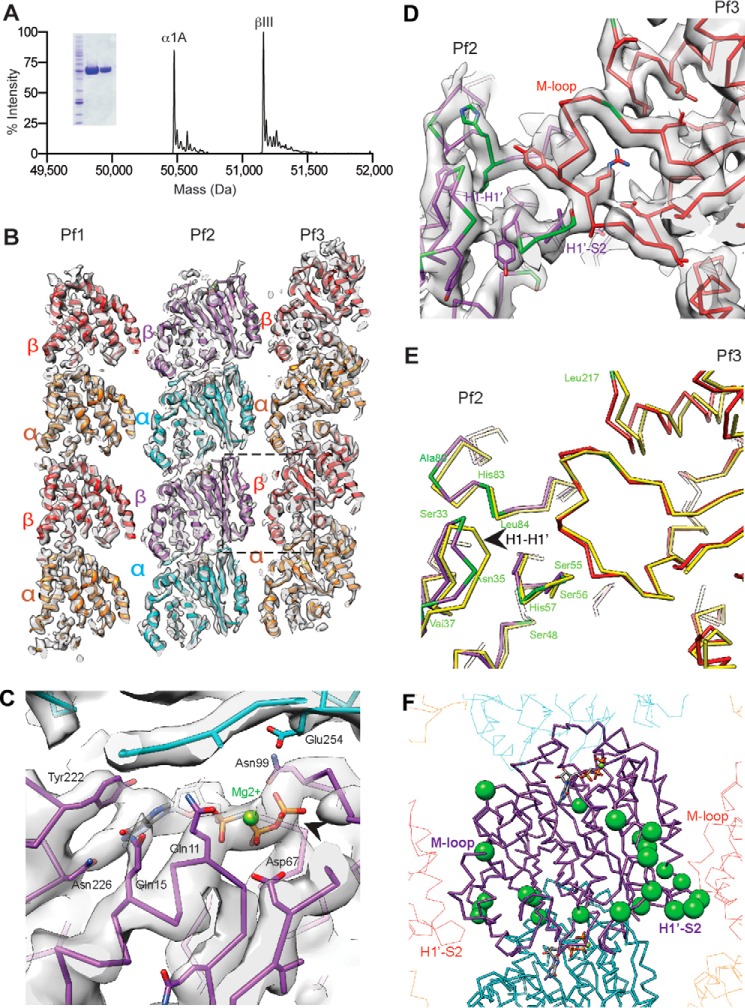
**Structure of unmodified single-isoform human α1A/βIII microtubules.** *A,* mass spectra and SDS-polyacrylamide gel (*inset*) of recombinant human α1A/βIII-tubulin purified to >99% homogeneity. The experimentally determined masses for α1A- and βIII-tubulin were 50,477.8 and 51,163.6 Da, respectively. The theoretical masses for α1A- and βIII-tubulin are 50,476.8 and 51,162.4 Da, respectively. *B,* cryo-EM map (4.2 Å resolution, 2.8 σ contour) and model of GMPCPP recombinant human α1A/βIII microtubules viewed from the lumen (three protofilaments shown). A central protofilament (Pf2) makes lateral contacts with adjacent protofilaments (Pf1 and Pf3); α-tubulin, *orange*, β-tubulin, *red* (Pf1, Pf3); α-tubulin, *cyan*; β-tubulin, *purple* (Pf2). *C,* E-site in βIII-tubulin shows clear density for GMPCPP and its three phosphate groups. *D,* model and map of the βIII-tubulin lateral interface (*boxed* and colored as in *B*). βIII-specific residues are in *green. E,* superposition of the α1A/βIII (colored as in *B*) and brain (PDB, 3JAT; atomistic models of brain microtubules use the βII isotype sequence because it constitutes ∼50% of these preparations (28, 44); *yellow*) microtubule structures; residues specific to βIII are in *green. F*, βIII sequence variability concentrates at the lateral interface. *Green spheres* denote residues that are different between the βIII and βII isotypes, the most abundant tubulin isoforms in brain tubulin preparations (10).

Codon-optimized genes for human α1A-tubulin (NP_001257328) with an internal His tag in the acetylation loop and a PreScission protease-cleavable C-terminally FLAG-tagged βIII-tubulin (NM_006077) were custom-synthesized by Integrated DNA Technologies and cloned into a pFastBac^TM^-Dual vector as described previously (16, 17). The internal His tag in α-tubulin allowed production of an α-tubulin ending in its natural C-terminal tyrosine (17, 18). Without an affinity-based selection for α-tubulin, the final sample contains ∼30% contamination with endogenous insect α-tubulin species that can be variable from construct to construct. The Bac-to-Bac System (Life Technologies, Inc.) was used to generate bacmids for baculovirus protein expression. High-Five or SF9 cells were grown to a density between 1.3 and 1.6 × 10^6^ cells/ml and infected with viruses at the multiplicity of infection of 1. Cultures were grown in suspension for 48 h, and cell pellets were collected, washed in PBS, and flash-frozen. Cells were lysed by gentle sonication in 1× BRB80 buffer (80 mm PIPES, pH 6.9, 1 mm MgCl_2_, 1 mm EGTA) with addition of: 0.5 mm ATP, 0.5 mm GTP, 1 mm PMSF, and 25 units/μl benzonase nuclease. The lysate was supplemented with 500 mm KCl and cleared by centrifugation (15 min at 400,000 × *g*). The crude supernatant (supplemented with 25 mm imidazole, pH 8.0) was loaded on a nickel-nitrilotriacetic acid column (Qiagen) equilibrated with high salt buffer (BRB80, 500 mm KCl, 25 mm imidazole). His-tagged tubulin was eluted with 250 mm imidazole in BRB80 buffer. The eluate was further purified on anti-FLAG G1 affinity resin (Gen Script). FLAG-tagged tubulin was eluted by incubation with FLAG peptide (GenScript) at 0.25 g/liter concentration followed by removal of the tag by PreScission protease. A final purification step was performed on a Resource Q anion exchange column (GE Healthcare) with a linear gradient from 100 mm to 1 m KCl in BRB80 buffer. Peak fractions were pooled and buffer-exchanged on a PD10 desalting column (GE Healthcare) equilibrated with BRB80, 20 μm GTP. Small aliquots of tubulin were frozen in liquid nitrogen and stored at −80 °C until use. The purified tubulin was subjected to ESI-TOF LC-MS analysis and detected no endogenous tubulin or post-translational modifications (Fig. 1*A*). The sensitivity of our mass spectrometric analyses is high enough to detect as little as 1% contaminating post-translationally modified tubulin species (17). The final yield is ∼1 mg of >99% recombinant isotypically pure αβ-tubulin per liter of SF9 cells.

##### Cryo-EM Sample Preparation and Data Collection

Recombinant human α1A/βIII-tubulin was polymerized at a final concentration of 2.5 mg/ml in BRB80 buffer (80 mm PIPES, 2 mm MgCl_2_, 1 mm EGTA, 1 mm DTT) with 1 mm GMPCPP^4^ or 2 mm GTP at 37 °C for 1 h. GMPCPP-bound microtubules were double-cycled by depolymerizing on ice and then repolymerized at 37 °C for 1 h with an additional 2 mm GMPCPP. Stabilized α1A/βIII microtubules were diluted in BRB20 (20 mm PIPES, 2 mm MgCl_2_, 1 mm EGTA, 1 mm DTT) to a final concentration of 2.5 μm. Human kinesin-3 motor domain (Kif1A, residues 1–361) (19) was diluted to 20 μm in BRB20 with 2 mm AMPPNP. The microtubules and motor were applied sequentially to glow-discharged C-flat^TM^ holey carbon grids (Protochips), and the sample was vitrified using a Vitrobot (FEI Co.). The presence of the kinesin motor domain allowed differentiation between α- and β-tubulin during processing. Images were collected with a DE20 direct electron detector (Direct Electron) on a FEI Tecnai G2 Polara operating at 300 kV with a calibrated magnification of ×52,117 corresponding to a final sampling of 1.22 Å/pixel. A total electron dose of ∼50 e^−^/Å^2^ over a 1.5-s exposure and a frame rate of 15 frames/s was used, giving a total of 23 frames at ∼2.2 electrons/frame. Dynamic microtubules grown from GMPCPP seeds were polymerized at 2 mg/ml for 30 min, kept at 37 °C throughout, and vitrified as above. Images were collected on a FEI Tecnai T12 operating at 120 kV using a 4096 × 4096-pixel CCD camera (Gatan Inc.).

##### Data Processing for Three-dimensional Reconstruction

**FIGURE 2.**
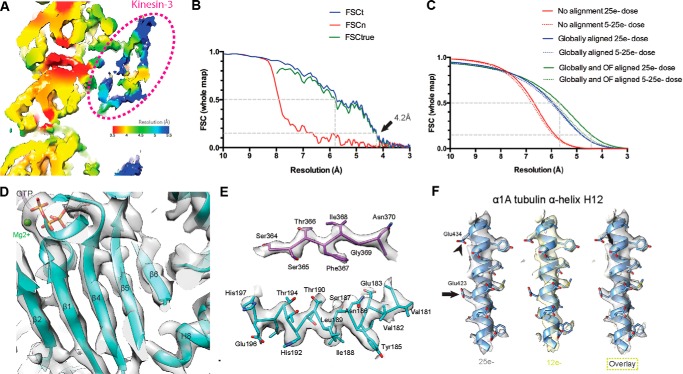
**Data processing, map quality, and resolution determination for cryo-EM reconstruction of recombinant human α1A/βIII microtubules.** *A,* local resolution estimates calculated using the Bsoft program blocres (51) were used to color the unfiltered whole reconstruction density. *Red* density corresponds to 3.5 Å resolution, with a continuum of colors indicating the resolution gradient, ending with *blue* at 5.5 Å resolution. Tubulin is at a higher resolution, ranging from ∼3.5 Å in central regions to ∼4.5 Å in a more flexible peripheral surface-exposed region. Although used for the initial alignment, kinesin-3 is less ordered (resolution of ∼5.5 Å) and is excluded from display items. *B,* Fourier shell correlation (*FSC*) curves. The gold standard noise-substitution test (26) on the whole microtubule + kinesin-3 map indicates no overfitting at high resolution and an overall resolution of 4.2 Å (*FSCtrue* at 0.143 cutoff). *C, R*_measure_ (52) fitted curves give the same resolution estimate. Global alignment of whole movie frames improved resolution dramatically, whereas local alignment using an optical flow technique (21) yielded further improvements, especially for frames from early dosing of the data most susceptible to beam-induced motion. *D,* higher resolution (<4 Å) in the tubulin dimer core is supported by clear density for the backbone and most side chains (see also *E*). *E,* representative density for a β-strand in β-tubulin (*top*) and an α-helix in α-tubulin (*bottom*). *F,* reconstructions from the first 12 e^−^/Å^2^ dose data (*yellow*) showed improved density for some side chains when compared with the 25 e^−^/Å^2^ dose data (*gray*), regardless of whether they were acidic. The highly negatively charged helix H12 of α-tubulin is shown. *Arrowheads* indicate acidic side chains that are notable for their different appearance in 12 and 25 e^−^/Å^2^ maps.

Individual ∼2.2 e^−^/Å^2^ frames were globally aligned using IMOD scripts (20) then locally aligned using the Optical Flow approach (21) implemented in Xmipp (22). The full dose of ∼50 e^−^/Å^2^ was used for particle picking and CTF determination in CTFFind3 (23), whereas ∼25 e^−^/Å^2^ was used in particle processing to center particles and determine their Euler angles. Euler angles and shifts determined using ∼25 e^−^/Å^2^ dose were used to generate reconstructions from either the first ∼25 or ∼12 e^−^/Å^2^ of the exposure. Kinesin-3 microtubules were manually boxed in Eman Boxer (24), serving as input for a set of custom-designed semi-automated single-particle processing scripts utilizing Spider and Frealign as described previously (25) with minor modifications. 10,164 particles or 142,296 asymmetric units were used in the final reconstruction, which was assessed for overfitting using a high resolution noise-substitution test (26). Using local resolution estimates determined with the blocres program in Bsoft, the reconstruction was sharpened with a *B* factor of −180 up to a resolution of 5.5 or 4 Å for visualization of kinesin or tubulin densities, respectively. The overall resolution of the reconstruction is 4.2 Å (FSCtrue, 0.143 criteria) (26) encompassing a resolution range of ∼3.5–5.5 Å. The best regions of the reconstruction are within the tubulin portion of the complex (Figs. 1*B* and 2) from which we built an α1A/βIII microtubule model. The quality of our reconstruction was sufficient to confirm that GMPCPP was found in the E-site (Fig. 1*C*) and GTP in the N-site.

##### Model Building and Refinement

The polypeptide model of the unmodified α1A/βIII-tubulin GMPCPP microtubule was built directly into density in Coot (27) using PDB 3JAT (28) as a starting model. The structure was refined under symmetry restraints in REFMAC version 5.8 (29). Secondary structure and reference restraints based on the high resolution tubulin crystal structure PDB 4DRX (30) were generated with ProSMART (31). Model building in Coot and refinement in REFMAC were repeated iteratively until the quality of the model and fit were optimized (supplemental Table 1).

##### In Vitro Microtubule Dynamics Assays

GMPCPP stabilized seeds were prepared as described (32). The GMPCPP seeds were immobilized in flow chambers using neutravidin as described previously (33). The final imaging buffer contained 1× BRB80 supplemented with 1 mm GTP, 100 mm KCl, 1% pluronic F-127, and oxygen scavengers prepared as described (34). An objective heater (Bioptechs) was used to warm the chamber to 30 °C. All chambers were sealed and allowed to equilibrate on the microscope stage for 5 min prior to imaging. Dark field images were acquired every 5 s for 30 min. For depolymerization rate measurements, the frame rate used was 40 frames/s. Imaging was performed on a Nikon Eclipse Ti-E equipped with a high NA dark field condenser, a ×100 adjustable iris objective and a Hamamatsu Flash4.0 version 2 camera with 2 × 2 binning. The final pixel size was 108 nm. Dark field illumination was provided by a Lumencor SOLA SE-II light engine. A Nikon GIF filter was used to protect the seeds from excessive photodamage.

##### Dynamic Parameter Measurements

Using ImageJ, kymographs were generated from dark field images. Kymographs were traced by hand, and dynamic parameters were calculated. Growth and depolymerization rates were determined from the slope of the growing or depolymerizing microtubule in the kymographs. Catastrophe frequency was determined as the number of observed catastrophes divided by the total time spent in the growth phase. Extremely rare rescue events were observed under our experimental conditions and thus were not quantified. Mean microtubule lifetime was calculated as the average time a microtubule spent in the growth phase before a catastrophe. Mean microtubule length was calculated as the average length a microtubule reached before a catastrophe. The probability of nucleation was determined by determining the percentage of seeds that nucleated in 30 min in a field of view. Dynamicity was determined as defined in Toso *et al.* (35) as the sum of total growth and shortening lengths divided by total time.

## Results

### 

#### 

##### Near Atomic Resolution Structure of Single-isoform Human α1A/βIII Microtubules

We selected for our study α1A/βIII-tubulin. βIII is a neuronal isoform that constitutes 25% of purified brain tubulin (10). It is expressed in non-neuronal tissues only during tumorigenesis (36, 37). It is also the most divergent of all β-tubulin isotypes. It is highly overexpressed in non-neuronal cells upon transformation and has been identified as a strong prognosticator of poor clinical outcomes (37). We expressed human α1A/βIII-tubulin in insect cells (16). Through a new double-selection strategy using affinity tags on both α- and β-tubulin, we produced >99% homogeneous, modification-free, single-isotype human αβ-tubulin, free of contamination from endogenous insect tubulins (Fig. 1*A* and see under “Experimental Procedures”) that is assembly-competent in the absence of stabilizing drugs like taxol and thus suitable for *in vitro* dynamics assays. Our tagging scheme generates an α-tubulin with a native C terminus and thus this recombinant tubulin is suitable for the investigation of the effects of the tubulin detyrosination/tyrosination cycle on intrinsic microtubule dynamics and those mediated by the modification-dependent recruitment of cellular effectors (38, 39).

To gain insight into the assembly properties of α1A/βIII recombinant tubulin, we determined the structure of α1A/βIII microtubules in complex with the GTP analog GMPCPP at near-atomic resolution using cryo-electron microscopy and single-particle image reconstruction (Figs. 1*B* and 2) (25). There is a resolution gradient in the reconstruction, with the best resolution (∼3.5 Å) within the body of the microtubule (encompassing a resolution range of ∼3.5–4.5 Å, Fig. 2*A*). The resolution range of the kinesin motor domain, used to facilitate reconstruction, is ∼4.5–5.5 Å. Overall, the reconstruction has a resolution of 4.2 Å (Fourier shell correlation, 0.143 criterion (26), encompassing a resolution range of ∼3.5–5.5 Å) (Fig. 2, *B* and *C*). The reconstruction shows clearly resolved β-sheets and α-helical pitch (Fig. 2, *D–F*). The majority (92%) of human α1A/βIII GMPCPP microtubules have 14 protofilaments, similar to brain GMPCPP microtubules (40). The tubulin monomer consists of a well folded globular core and highly negatively charged and flexible C-terminal tails (41). The C-terminal tails are the locus of the greatest chemical heterogeneity in tubulin. They appear disordered in all microtubule structures to date either because (i) they have no unique well defined conformation or (ii) defined conformations unique to particular isoforms or post-translationally modified forms are lost during the iterative averaging used in EM reconstructions due to the high heterogeneity of these tails in brain tubulin samples. Despite the chemical homogeneity of our sample, there is no density attributable to them, indicating that they are intrinsically disordered unless engaged by an effector as seen for the tubulin tyrosine ligase like 7 glutamylase or the NDC80 complex (42–44).

**FIGURE 3.**
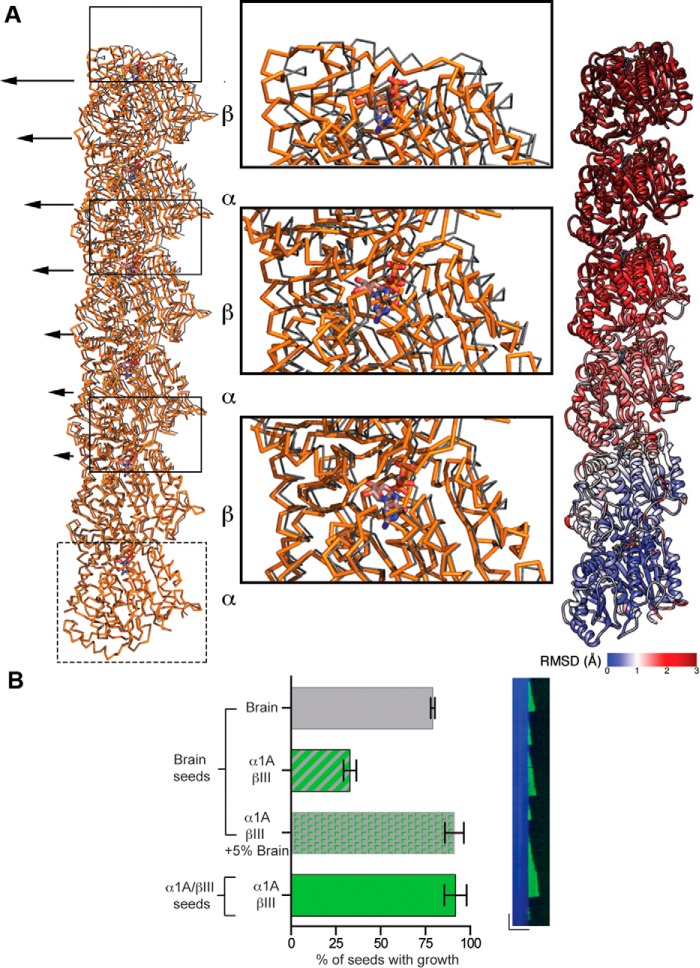
**Comparison between α1A/βIII and mosaic brain 14 protofilament microtubule structures.** *A, left panel,* dimer displacement compared with the structure of mosaic brain microtubules PDB 3JAT (28) as viewed from the microtubule lumen. The *boxed* α1A-tubulin protomer from the α1A/βIII structure (*orange C*α *trace*) was superimposed on the α-tubulin protomer from the brain microtubule structure (*gray C*α *trace*). *Arrows* indicate the gradual increase in displacement of the α1A/βIII heterodimers as one advances toward the plus-end of the protofilament. The GTP and GMPCPP in the N-site of α-tubulin and the E-site of β-tubulin are shown as *ball-and-stick. Middle panel,* zoomed in view of regions highlighted by *boxes* in the *left panel* showing details of the displacement between the dimers from the recombinant α1A/βIII and brain microtubule structures; *Right panel,* three α1A/βIII heterodimers within one protofilament colored according to main chain displacement from the brain microtubule structure. *B, left panel,* percentage of seeds that nucleate microtubules at 6 μm tubulin. Brain, α1A/βIII, α1A/βIII + 5% brain tubulin elongated from brain seeds and α1A/βIII-tubulin elongated from α1A/βIII seeds. More than 100 seeds across multiple chambers were counted for these measurements. *Right panel,* kymograph of microtubule growth for recombinant α1A/βIII at 5.7 μm supplemented with 5% Hilyte 488 brain tubulin (0.3 μm) from brain GMPCPP seeds showing incorporation of the brain tubulin into the α1A/βIII lattice. *Horizontal* and *vertical scale bar,* 5 μm and 2 min, respectively.

Consistent with the high sequence conservation of the tubulin body, our structure is similar to that of heterogeneous mosaic mammalian brain GMPCPP microtubules, and the overall conformation of the tubulin dimers in our reconstruction is consistent with a GTP-like extended conformation (Fig. 1*C*) (28). The backbone root mean square deviation of our tubulin dimer model overlaid on that of the recently published structure of mammalian heterogeneous brain GMPCPP 14 protofilament microtubules is <2 Å. A difference in the tubulin repeat distance is observed between α1A/βIII and brain microtubules as follows: 82.7 ± 0.2 *versus* 83.1 ± 0.0 Å measured from the EM reconstruction (*i.e.* model-independent); 82.6 *versus* 83.2 Å measured by comparing models, for α1A/βIII and brain microtubules, respectively (28, 45). However, the tubulin repeat distance for the recombinant α1A/βIII microtubules (∼82.7 Å) is roughly comparable with the repeat distance for heterogeneous brain GMPCPP microtubules (∼83 Å), which is more extended than that of the GDP state (∼81.5 Å) (28, 45). Nevertheless, we find two subtle differences that have the potential to impact polymerization dynamics. First, the loop connecting helices H1 and H1′ in β-tubulin shifts ∼3 Å away from the H1′-S2 loop, which makes lateral contacts with the M-loop (microtubule loop) of the neighboring dimer (Fig. 1, *D* and *E*). The M-loop is a sequence element crucial to lateral contacts between adjacent protofilaments. Strikingly, the H1′-S2, H2-S3, and M-loops are a hot spot of sequence variation across β-tubulin isoforms (Fig. 1*F*), consistent with the structural plasticity we observe at this interface. Second, when one α-protomer each of brain GMPCPP and recombinant α1A/βIII GMPCPP microtubule protofilaments are superimposed, a clear displacement of successive recombinant α1A/βIII dimers becomes apparent (Fig. 3*A*). This propagates from the exchangeable GTP-site (E-site) and βIII-tubulin longitudinal interface and results in a progressive stagger that increases with each dimer along the protofilament, such that the first neighboring dimer is offset by 1.7 Å (all Cα root mean square deviation), the second by 3.4 Å, and so on. Together, these relatively subtle structural differences could contribute to differences in dynamic properties. Interestingly, we find that at 6 μm α1A/βIII-tubulin, 92% of α1A/βIII GMPCPP seeds nucleate microtubules but only 33% brain seeds nucleate α1A/βIII microtubules (Fig. 3*B*), suggestive of lattice mismatch effects between the brain microtubule seed and the lattice parameters of the growing α1A/βIII microtubule. This is consistent with the subtle structural differences between α1A/βIII and heterogeneous brain microtubules that we identified (Figs. 1, *D* and *E,* and 3*A*). Unexpectedly, robust growth off brain seeds at 6 μm α1A/βIII could be rescued (from 33 to 91%) if as little as 5% brain tubulin was added (Fig. 3*B*). Thus, a small level of tubulin heterogeneity can alleviate the nucleation defect that arises from the potential mismatch between the lattices of the two microtubule types. Our finding has intriguing consequences for the nucleation *in vivo* of microtubules composed of mixtures of tubulin isoforms.

##### In Vitro Dynamics of Single-isoform α1A/βIII-tubulin

**FIGURE 4.**
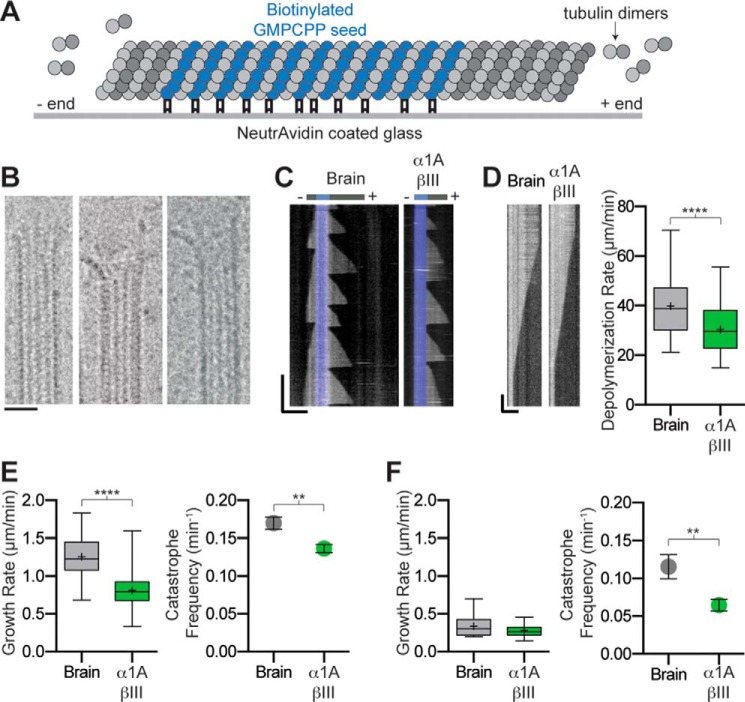
**Dynamic parameters of recombinant human α1A/βIII microtubules.** *A,* schematic of assay design (see under “Experimental Procedures”). *B,* micrographs of representative dynamic α1A/βIII microtubule ends. *Scale bar,* 20 nm. *C,* kymographs showing typical microtubule growth for brain and recombinant α1A/βIII-tubulin at 9 μm. *Blue* marks the GMPCPP seed. *Horizontal* and *vertical scale bars,* 5 μm and 5 min, respectively. *D, left panel*, kymographs showing a typical depolymerization event for brain and α1A/βIII microtubules. *Horizontal* and *vertical scale bar,* 5 μm and 2 s, respectively. *Right panel*, Tukey plot showing plus-end depolymerization rates at 9 μm tubulin; *n* = 55 and 58 events for brain and α1A/βIII microtubules, respectively. *E,* plus-end dynamics of brain and α1A/βIII-tubulin at 9 μm tubulin. *Left panel*, box-whisker plot (*whiskers* indicate minimum and maximum) showing growth rates; *n* = 255 and 504 events for brain and α1A/βIII-tubulin, respectively. *Right panel*, catastrophe frequencies; *n* = 48 and 167 microtubules for brain and α1A/βIII-tubulin, respectively. *F,* minus-end dynamics of brain and α1A/βIII-tubulin at 9 μm tubulin. *Left panel*, box-whisker plot (*whiskers* indicate minimum and maximum) showing growth rates; *n* = 32 and 25 events for brain and α1A/βIII-tubulin, respectively. *Right panel*, catastrophe frequencies; *n* = 7 and 16 microtubules for brain and α1A/βIII-tubulin, respectively. *Error bars* represent S.E. ** and ****, *p* values < 0.01 and < 0.0001, respectively determined by unpaired *t* test.

To determine dynamic parameters of single-isoform α1A/βIII-tubulin, we performed label-free *in vitro* dynamic assays using dark field microscopy (Fig. 4 and supplemental Movies 1 and 2) (46) so that our dynamic parameters are not confounded by effects arising from the addition of fluorescently labeled brain tubulin to the otherwise homogeneous microtubules. The α1A/βIII microtubules have the typical end appearance observed for brain microtubules consisting of a mixture of short sheet-like and blunter structures (Fig. 4*B*) (47). To quantify their dynamics, we generated kymographs from time-lapse imaging of dynamic microtubule assays (Fig. 4*C*). The growth rates at the plus-end are 35% slower when compared with those of heterogeneous brain tubulin, whereas the minus-end growth rates are statistically indistinguishable. Consistent with this, the on-rate of α1A/βIII-tubulin at the plus-end is 1.8 dimers s^−1^ μm^−1^ compared with the 3.6 dimers s^−1^ μm^−1^ for brain tubulin (our measurements for brain microtubules are similar to those reported in Ref. 48). Dark field imaging allows data collection at the high frame rates needed to determine microtubule depolymerization rates with high accuracy (“Experimental Procedures” and see supplemental Movie 3). These measurements revealed that α1A/βIII microtubules depolymerize slower than brain microtubules (30.5 ± 1.3 μm/min *versus* 39.9 ± 1.5 μm/min; Fig. 4*D*). This suggests that microtubules with different chemical compositions (isoform or post-translational modifications) have the potential to generate different end depolymerization forces that could be harnessed to move cargo in the cell, such as chromosomes during cell division (49).

The catastrophe (the transition between growth and shrinkage) frequency of recombinant microtubules is slightly reduced by 20 and 44% at the plus- and minus-ends, respectively, when compared with heterogeneous brain tubulin (Fig. 4, *E* and *F*). Interestingly, although 46% of brain microtubule exhibit growth at their minus ends, fewer than 7% of recombinant microtubules display minus-end dynamics under our assay conditions. Early studies reported faster polymerization rates for αβIII-tubulin (α denotes here an unknown mixture of α-tubulin isoforms) immunopurified from brain tubulin preparations than for brain tubulin (15). Those studies also found that αβIII-tubulin immunopurified from brain tubulin preparations had higher dynamicity than brain tubulin, although our measurements with recombinant α1A/βIII show lower dynamicity for this species than for brain microtubules (1.31 ± 0.05 μm/min *versus* 2.30 ± 0.07 μm/min for α1A/βIII and brain, respectively; “Experimental Procedures”). However, it is important to note that the tubulin used in these earlier studies had an unknown α-tubulin composition and a poorly defined mixture of diverse post-translational modifications, unlike our recombinant tubulin, which contains a single α- and β-tubulin isoform and is unmodified (Fig. 1*A* and “Experimental Procedures”). It is unclear at this point whether the subtle differences in dynamics we observe between the recombinant α1A/βIII microtubules and heterogeneous mosaic brain microtubules are due to isoform differences, purification method, and/or the abundant and diverse post-translational modifications found on brain microtubules. Future studies with recombinantly expressed isoforms and quantitatively defined post-translationally modified tubulin using the expression and purification system described here will shed light on their individual contributions to dynamic instability parameters.

## Discussion

Using our dual-tag purification system for recombinant tubulin, we report for the first time the structure and *in vitro* dynamics parameters for isotypically pure human unmodified microtubules, an essential and important initial step in quantitatively establishing the correlates between sequence and dynamics for mammalian microtubules. The dual-tag selection system is necessary as a single-tag purification strategy results in significant levels of contamination with endogenous tubulin (∼30% of insect α-tubulin if α-tubulin is not selected via an affinity tag). Thus, our tagging and purification strategy allows the characterization of both α- and β-tubulin engineered constructs. The majority of *in vitro* dynamics studies presently performed use heterogeneous mosaic brain microtubules with isoform composition and post-translational modifications different from those found *in vivo*, for example in an epithelial cell or the axonal or dendritic compartment of a neuron. A recent study revealed different activities of the *Saccharomyces cerevisiae* Stu2p on yeast microtubules compared with heterogeneous brain microtubules (50), indicating the importance of examining the effects of regulators with the physiologically relevant tubulin substrate. Our study establishes a system to examine the dynamics of mammalian microtubules with well defined tubulin species and opens the way to study tubulin isoform-specific effects of microtubule-associated proteins and motors and to uncover the tubulin sequence elements critical for their recruitment and activation.

## Author Contributions

A. R.-M. conceived the project. A. V. and J. O. S. performed and analyzed the dynamics assays. J. A. determined EM structure, and A. S. purified recombinant tubulin. All authors interpreted data. A. R.-M. wrote the manuscript with contributions from A. V., J. O. S., J. A., and C. A. M.

## Supplementary Material

Supplemental Data

## Footnotes

4 The abbreviations used are:

GMPCPP: guanylyl-(α,β)-methylene-diphosphonate
AMPPNP: 5′-adenylyl-β,γ-imidodiphosphate
PDB: Protein Data Bank.
