# Supplementary material for: Structure and Dynamics of Single-isoform Recombinant Neuronal Human Tubulin
Source: J Biol Chem. 2016 Apr 25;291(25):12907–15. doi: 10.1074/jbc.C116.731133 (PMC4933209; doi:10.1074/jbc.C116.731133)
Supplement: Supplemental Data [file supp_291_25_12907__index.html]

Structure and Dynamics of Single-isoform Recombinant Neuronal Human Tubulin — Structure and Dynamics of Single-isoform Recombinant Neuronal Human Tubulin — REPORT: Structure and Dynamics of Human α1A/βIII-tubulin — Supplemental Data 

# Structure and Dynamics of Single-isoform Recombinant Neuronal Human Tubulin

## Supplemental Data

- Supplemental Table 1 (.pdf, 83 KB) - Supplemental Table 1 and Movie Legends
- Supplemental Movie 1 (.avi, 57.0 MB) - Dynamics of recombinant human α1A/βIII microtubules imaged by darkfield microscopy.
- Supplemental Movie 2 (.avi, 47.0 MB) - Dynamics of heterogeneous brain microtubules imaged by darkfield microscopy.
- Supplemental Movie 3 (.avi, 2.0 MB) - Depolymerizing human α1A/βIII microtubule imaged by darkfield microscopy at high temporal resolution.
