# Supplementary material for: Structure and Dynamics of Single-isoform Recombinant Neuronal Human Tubulin
Source: J Biol Chem. 2016 Apr 25;291(25):12907–15. doi: 10.1074/jbc.C116.731133 (PMC4933209; doi:10.1074/jbc.C116.731133)
Supplement: Supplemental Data [file 10.1074_C116.731133_jbc.C116.731133-1.pdf]

Structure and dynamics of single-isoform recombinant neuronal human tubulin

Annapurna Vemu[1§], Joseph Atherton[2§], Jeffrey O. Spector[1§], Agnieszka Szyk[1], Carolyn A. Moores[2,*] and Antonina Roll-Mecak[1,3,*]

SUPPLEMENTAL TABLE 1. Refinement statistics and model geometry
Refinement statistics and model geometry from REFMAC v5.8 (29) and MolProbity (51).

Resolution for refinement	4 Å
Map sharpening B factor	-180
$FSC_{average}$[a]	0.79
Rms deviations (bonds)($Å^2$)	0.008
Rms deviations (angles)($Å^2$)	1.438
Molprobity Score	1.75 (100th percentile)
Clashscore, all atoms	7.39 (100th percentile)
Poor rotamers (%)	1.18%
Ramachandran plot favoured (%)	95.75%
Ramachandran outliers (%)	0.12%

[a]aFSCaverage = $\Sigma(N_{shell}\ FSC_{shell})/\ \Sigma(N_{shell})$, where FSC_{shell} is the FSC in a given shell, N_{shell} is the number of structural factors in the shell. $FSC_{shell} = \Sigma(F_{model}\ F_{EM})/(\surd\Sigma(|F|2_{model}))\surd\Sigma(F^2{}_{EM}))$

SUPPLEMENTAL MOVIE 1

Dynamics of recombinant human α1A/βIII microtubules imaged by darkfield microscopy.
Dynamic recombinant human α1A/βIII microtubules at 9 μM tubulin imaged by darkfield microscopy. Blue marks the GMPCPP seed. Recorded at 12 frames per minute, played back at 30 frames per second.

SUPPLEMENTAL MOVIE 2

Dynamics of heterogeneous brain microtubules imaged by darkfield microscopy.
Dynamic brain microtubules at 9 μM tubulin imaged by darkfield microscopy. Blue marks the GMPCPP seed. Recorded at 12 frames per minute, played back at 30 frames per second.

SUPPLEMENTAL MOVIE 3

Depolymerizing human α1A/βIII microtubule imaged by darkfield microscopy at high temporal resolution. Scale bar, 2.5 μm. Recorded at 40 frames per second played back at 30 frames per second.
